# A clinical decision support system promotes the appropriate use of drugs in hospitalized patients with kidney impairment

**DOI:** 10.1186/s40780-025-00431-8

**Published:** 2025-04-03

**Authors:** Akihiro Sonoda

**Affiliations:** Department of Pharmacy, Izumi Regional Medical Center, 4513 Akasegawa, Akune, 899 - 1611 Japan

**Keywords:** Acute kidney injury, Clinical decision support system, Chronic kidney disease, Kidney impairment, Hospitalized patients, Nephrotoxic drugs, Pharmacist, Physician, Renally excreted drugs

## Abstract

The number of individuals with chronic kidney disease (CKD) is increasing worldwide, including in Japan. Patients with advanced CKD are at an increased risk of serious adverse drug events associated with hospitalization, life-threatening complications, and death.

It is necessary to adjust the dosage of renally excreted drugs according to kidney function in patients with CKD. In addition, elderly patients and those with impaired kidney function are also at high risk of drug-induced nephrotoxicity due to nephrotoxic drugs, and special attention should be paid to changes in kidney function before and after administration. Hospitalized patients are more susceptible to acute kidney injury than outpatients, and care must be taken when administering renally excreted or nephrotoxic drugs. Clinical decision support systems (CDSSs) play an important role in preventing overdosage of renally excreted drugs and avoiding the inappropriate use of nephrotoxic drugs.

This review discussed the effectiveness, issues, and potential of CDSSs for physicians’ prescriptions and pharmacists’ prescription audits before hospitalized patients with kidney impairment are administered renally excreted drugs or nephrotoxic drugs, and the follow-up of patients receiving them. Although inappropriate prescriptions of renally excreted drugs due to alerts to prescribers were reduced, prescribers may have ignored interruption alerts. Therefore, the acceptance rate of alerts by prescribers can be improved by minimizing interruptions to the prescriber workflow, specifying only high-severity alerts, and automatically inputting the dosage, administration frequency, and administration duration according to kidney function when the prescriber selects a drug when entering a prescription. Prescription audits by pharmacists using electronic alerts from the CDSS and dosage confirmation sheets were effective in preventing overdosing of renally excreted drugs. In addition, pharmacist interventions for patients at risk of acute kidney injury (AKI) using CDSS alerts may be useful in preventing a decrease in kidney function and the onset of AKI due to nephrotoxic drugs. Although the usefulness of CDSSs may be further improved in the future, further evaluation and improvement of CDSSs are required.

## Background

Chronic kidney disease (CKD) is a common condition in Japan [1]. As CKD progresses, the risk of hospitalization, cardiovascular events, and death increases [2]. The number of people suffering from CKD is increasing worldwide [3], including in Japan [4, 5]. Pharmacokinetics are altered in patients with CKD [6, 7] and patients with reduced kidney function, such as those with CKD, have a higher incidence of adverse events caused by drugs excreted renally [8]. In particular, patients with advanced CKD are at high risk of serious adverse drug events associated with hospitalization, life-threatening complications, and death [9]. Therefore, the dosage of these drugs must be adjusted according to the kidney function. In addition, elderly patients and those with kidney impairment are at a high risk of drug-induced kidney injury caused by nephrotoxic drugs [10, 11], and special attention should be paid to changes in kidney function before and after drug administration.

**Fig. 1:**
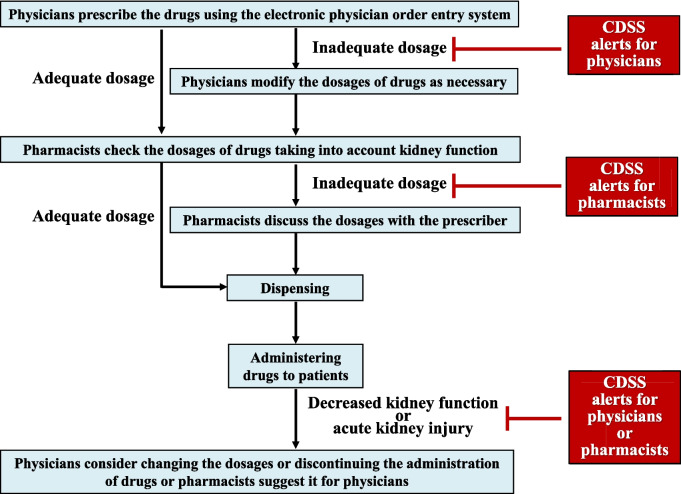
Checkpoints to prevent overdosage of renally excreted drugs and avoid the inappropriate use of nephrotoxic drugs. Before renally excreted drugs or nephrotoxic drugs are administered to a patient, there are two checkpoints. First, physicians prescribe the drugs using the electronic physician order entry system. If the dosage is inadequate, a CDSS alert is sent to physicians, and they modify the dosages of drugs as necessary. Second, pharmacists check the dosages of drugs taking into account kidney function. If the dosage is inadequate, a CDSS alert is sent to pharmacists, and they discuss the dosages with the prescriber and dispense drugs. Then, the drug is administered to a patient. If a decreased kidney function or acute kidney injury occurs during drug administration, a CDSS alert is sent to physicians or pharmacists. Physicians consider changing the dosages or discontinuing the administration of drugs or pharmacists suggest it for physicians. CDSS: clinical decision support system

In a study aimed at developing and validating a risk prediction model for acute kidney injury (AKI) in outpatient care, the incidence of outpatient AKI was approximately 2.4% in the validation cohort [12]. In contrast, a meta-analysis reported that 21.6% of adults worldwide developed AKI during hospitalization [13]. These findings suggest that hospitalized patients are at a higher risk of developing AKI than outpatients. Therefore, hospitalized patients should be careful about declining kidney function, in AKI. However, it is difficult to prescribe renally excreted drugs or nephrotoxic drugs considering kidney function [14–18]. In addition, whether pharmacists can properly audit prescriptions containing renally excreted drugs or nephrotoxic drugs is influenced by their awareness and knowledge [19–21]. Therefore, systems that support prescribers and pharmacists are needed to ensure the appropriate use of these drugs in hospitalized patients with kidney impairment. Clinical decision support systems (CDSSs) play an important role in preventing overdosage of renally excreted drugs and avoiding the inappropriate use of nephrotoxic drugs [22]. Before renally excreted drugs or nephrotoxic drugs are administered to a patient, there are two checkpoints: (1) physicians prescribe renally excreted drugs or nephrotoxic drugs while considering kidney function, and (2) pharmacists audit their dosages while considering kidney function (Fig. 1). In addition, it is necessary to monitor the patient’s kidney function after drug administration and intervene early if kidney damage occurs (Fig. 1).

In this review, the author discusses the effectiveness, issues, and potential of CDSSs for physicians’ prescriptions and pharmacists’ prescription audits before hospitalized patients with kidney impairment are administered renally excreted drugs or nephrotoxic drugs, and the follow-up of patients receiving them.

## Physicians’ approaches to promote the appropriate use of drugs using CDSSs when entering prescriptions

Order entry by physicians is the first checkpoint in preventing overdosing on renally excreted drugs (Fig. 1). Below, this review describes physicians’ approaches to promote the appropriate use of drugs using CDSSs when entering prescriptions (Table 1). Chertow et al. developed a system that notified prescribers of the dosage and dosage intervals based on kidney function when entering a prescription [23]. A total of 7490 hospitalized patients had an estimated creatinine clearance (eCCr) < 80 mL/min. In this group, 97,151 alerts for kidney function were generated, of which 14,440 (15%) led to computerized modifications of at least one dosing parameter based on kidney function. The rates of appropriate prescriptions according to kidney function during the intervention and control periods were 67% and 54% (*p* < 0.001) by dose and 59% and 35% (*p* < 0.001) by frequency, respectively. Terrell et al. created a decision support system that provided dosage recommendations for targeted medications for patients in an academic emergency department when the patient’s eCCr level was below the dosage adjustment threshold when physicians entered the prescription [24]. Physicians in the intervention group were provided with 73 alerts, 31 (42%) of which did not result in a dose reduction appropriate for kidney function, whereas physicians in the control group were provided with 46 alerts, 34 (74%) of which did not result in a dose reduction appropriate for kidney function (*p* = 0.001). Awdishu et al. implemented a system that alerted prescribers to reduce dosage based on kidney function when they entered a prescription in ambulatory and acute settings [25]. The rates of dose adjustment based on kidney function due to alerts at the time of prescription entry in the intervention and control groups were 55.6% (130/234) and 21.5% (52/242), respectively, and were significantly higher in the intervention group (*p* < 0.0001). Thus, although CDSSs can reduce the number of inappropriate prescriptions for renally excreted drugs, the rate of inappropriate prescriptions remains high. One reason for this is that prescribers’ acceptance of CDSS alerts is low and they are often ignored [26, 27]. Low-specificity alerts and vague alerts that do not provide clear information about the reason for the alert are associated with alert fatigue [28], which may lead to low acceptance of CDSS alerts for prescribers. Cho et al. investigated physician behavior in response to alerts about nephrotoxic medications in outpatient settings [29]. In this study, a total of 4120 alerts were sent by 584 prescribers, of which 78.2% (3221) were ignored. Of the 289 ignored alerts by 35 prescribers, 85 (29.4%) prescriptions were judged to be appropriate. The majority of reasons given for why ignoring the alert was appropriate were that “the patient has tolerated the medication in the past.” or “there is new evidence supporting this type of treatment”. However, of the 289 ignored alerts, 204 (70.6%) were inappropriately ignored. Compared with appropriate alert ignoring, inappropriate alert ignoring is more likely to cause adverse drug events [30]. Therefore, CDSSs need to be improved to prevent prescribers from ignoring important alerts and to increase prescribers’ acceptance of alerts. Shah et al. developed a selective drug alert system suitable for outpatient care to minimize workflow interruptions and improve prescribers’ acceptance of drug alerts by designating only high-severity alerts among those that interrupted the prescriber’s workflow [31]. Of the 5182 drug alerts received, 67% were accepted. The highest acceptance rate was for medication duplication alerts (77%) followed by drug disease alerts (53%). Thus, minimizing interruptions to the prescriber’s workflow and designating only high-severity alerts can improve the prescribers’ acceptance of drug alerts and reduce inappropriate prescriptions. Hirsch et al. created a system that automatically input the dosage, frequency, and duration of administration according to kidney function when prescribers select a drug upon entering the prescription [32]. Of the 44,267 automated recommended orders, 42,670 (96%) were accepted by prescribers. Choi et al. examined the impact of pharmacists’ interventions on prescribers’ acceptance of CDSS alerts [33]. The presence of designated pharmacists increased the acceptance rate of alerts (odds ratio 1.353, *p* = 0.0272). Thus, by continually evaluating inappropriate prescriptions and physician acceptance rates, striving to improve the CDSS, and having pharmacists intervene, the CDSS may be more effectively promoted.

**Table 1:** Summary of physicians’ approaches to promote the appropriate use of drugs using CDSSs when entering prescriptions

References	System features	Key results
Chertow et al [23]	A system notified prescribers of the dosage and dosage intervals based on kidney function when entering a prescription for hospitalized patients	The rates of appropriate prescriptions according to kidney function during the intervention and control periods were 67% and 54% (*p* < 0.001) by dose and 59% and 35% (*p* < 0.001) by frequency, respectively
Terrell et al. [24]	A system provided dosage recommendations for targeted medications for patients in an academic emergency department when the patient’s eCCr level was below the dosage adjustment threshold when physicians entered the prescription	Physicians in the intervention group were provided with 73 alerts, 31 (42%) of which did not result in a dose reduction appropriate for kidney function, whereas physicians in the control group were provided with 46 alerts, 34 (74%) of which did not result in a dose reduction appropriate for kidney function (*p* = 0.001)
Awdishu et al. [25]	A system alerted prescribers to reduce dosage based on kidney function when they entered a prescription in ambulatory and acute settings	The rates of dose adjustment based on kidney function due to alerts at the time of prescription entry in the intervention and control groups were 55.6% (130/234) and 21.5% (52/242), respectively, and were significantly higher in the intervention group (*p* < 0.0001)

*CDSS* clinical decision support system, *eCCr* estimated creatinine clearance

The utility of CDSSs in alerting physicians to patients with AKI has also been studied [34–37]. However, few studies have evaluated the utility of CDSSs in alerting physicians to avoid nephrotoxic drugs in patients at risk of AKI. Koyner et al. developed an AKI risk prediction algorithm using electronic health record data from ward patients to identify patients at risk of AKI in general wards before serum creatinine levels increase [38]. Of the 202,961 patients in this study, 17,541 (8.6%) developed AKI and 1242 (0.6%) progressed to AKI stage 3. The areas under the receiver operating characteristic (ROC) curve for the model, with only serum creatinine and blood urea nitrogen (BUN) were 0.69 (95% confidence interval (CI), 0.68–0.69) for AKI stage 1 and 0.74 (95% CI, 0.73–0.74) for AKI stage 3. The areas under the ROC curve for the final model including serum creatinine and BUN with demographics, vital signs, and laboratory tests (E-STOP-AKI) were 0.74 (95% CI, 0.74–0.74) for AKI stage 1 and 0.83 (95% CI, 0.83–0.84) for AKI stage 3. Thus, E-STOP-AKI may be useful in predicting AKI. Therefore, further studies are required to develop and evaluate systems that would alert prescribers when nephrotoxic medications are prescribed to patients identified by E-STOP-AKI as being at high risk for AKI.

## Pharmacists’ approaches to promote the appropriate use of medicines using CDSSs when auditing prescriptions

Pharmacists were involved in the final dosage decision (Fig. 1) and contributed to the appropriate use of renally excreted and nephrotoxic drugs through prescription audits and suggestions [39–45]. This review introduces reports on the use of CDSSs by pharmacists to promote the appropriate use of renally excreted drugs (Table 2). Bhardwaja et al. implemented a system that warned pharmacists when the dosage exceeded kidney function-based values when dispensing renally excreted drugs and evaluated the usefulness of the system in 3025 intervention groups and 3100 control groups that did not use the system in an ambulatory setting [46]. The rates of dosage errors in the intervention and control groups were 33% and 49%, respectively, and those in the intervention group were significantly lower (*p* < 0.001). Díaz et al. developed a system that allowed pharmacists in a tertiary teaching hospital to identify patients with reduced kidney function and medications that may require dosage modifications based on kidney function [47]. The rate of appropriate prescriptions based on kidney function increased significantly from 65 to 86% before and after the intervention, respectively (*p* < 0.001). Niedrig et al. developed a system to prospectively identify metformin prescriptions when the estimated glomerular filtration rate was less than 60 mL/min in a tertiary care hospital [48]. The system sent real-time electronic alerts to clinical pharmacologists and pharmacists, who verified the case in electronic medical records and, if necessary, confirmed the dose with the prescriber. Clinical pharmacologists and pharmacists who received electronic alerts contacted the prescriber to request the reduction or discontinuation of metformin in 240 patients. This suggestion was accepted in 191 patients and may help prevent metformin overdosage. Vogel et al. reported that the rate of dosage errors was 0.36% when using an outpatient system that alerted pharmacists when they dispense prescriptions for medications that require kidney dose adjustment or are contraindicated at certain kidney function thresholds [49]. Thus, CDSSs, which inform pharmacists about dosage issues at the time of dispensing, are thought to contribute to preventing the overdosage of renally excreted drugs. However, CDSSs have certain limitations in terms of their use, lack of transportability and interoperability, and cost of introducing and maintaining them [50]. Studies have focused on the use of prescriptions to avoid these issues [42, 43]. Ishikawa et al. developed a system that automatically printed a direct oral anticoagulant (DOAC) check sheet when DOACs were prescribed to hospitalized patients [42]. The number of inquiries from pharmacists to physicians regarding DOAC prescriptions, such as dosage, before the use of the DOAC check sheet was four out of 642 prescriptions (0.6%), but after its introduction, this number increased significantly to 21 out of 905 prescriptions (2.3%) (*p* = 0.0089), which is thought to prevent DOAC overdosage. The author implemented an in-hospital prescription checking system (PCS) for hospitalized patients that involves: (1) adding the label “renal” before the name of renally excreted drugs on the prescription; (2) adding the patient’s estimated kidney function level on the prescription; and (3) using a check sheet for the dosages based on kidney function for the target drugs (allopurinol, cibenzoline, famotidine, and pilsicainide) [43]. When a hospital prescription included the target drug, a check sheet based on the patient’s kidney function was automatically printed, and pharmacists checked the dosages of the renally excreted drugs during dispensing. If the target drug dosage was appropriate, the drugs were dispensed. If the dosage of the target drug exceeded the standard values for kidney function, pharmacists asked the prescriber about the prescription contents before dispensing the drugs. The overall dosage error rate for the four target drugs was 26% before PCS implementation, which decreased significantly to 3% after PCS implementation (*p* < 0.001). Thus, systems using prescriptions contribute to preventing the overdose of renally excreted drugs. Furthermore, the concept of a prescription system is simple and can be implemented in several facilities. However, when the target drugs were prescribed, a check sheet was printed regardless of whether the dosages were appropriate, which could place a burden on pharmacists [51]. Therefore, the system can be further developed by optimizing cases in which a check sheet is issued, such as when there is a problem with dosages according to kidney function, or when AKI or worsening kidney injury is suspected.

**Table 2:** Summary of pharmacists’ approaches to promote the appropriate use of medicines using CDSSs when auditing prescriptions

References	System features	Key results
Bhardwaja et al. [46]	A system warned pharmacists when the dosage exceeded kidney function-based values when dispensing renally excreted drugs	The rates of dosage errors in the intervention and control groups were 33% and 49%, respectively, and those in the intervention group were significantly lower (*p* < 0.001)
Díaz et al. [47]	A system allowed pharmacists in a tertiary teaching hospital to identify patients with reduced kidney function and medications that may require dosage modifications based on kidney function	The rate of appropriate prescriptions based on kidney function increased significantly from 65 to 86% before and after the intervention, respectively (*p* < 0.001)
Niedrig et al [48]	A system prospectively identified metformin prescriptions when the estimated glomerular filtration rate was less than 60 mL/min in a tertiary care hospital. The system sent real-time electronic alerts to clinical pharmacologists and pharmacists, who verified the case in electronic medical records and, if necessary, confirmed the dose with the prescriber	Clinical pharmacologists and pharmacists who received electronic alerts contacted the prescriber to request reduction or discontinuation of metformin in 240 patients. This suggestion was accepted in 191 patients
Vogel et al. [49]	An outpatient system alerted pharmacists when they dispense prescriptions for medications that require kidney dose adjustment or are contraindicated at certain kidney function thresholds	The rate of dosage errors was 0.36%
Ishikawa et al. [42]	A system automatically printed a DOAC check sheet including the information on dosages according to kidney function and cut-off values ​​for contraindications due to kidney function when DOACs were prescribed to hospitalized patients	The number of inquiries from pharmacists to physicians regarding DOAC prescriptions, such as dosage, before the use of the DOAC check sheet, was four out of 642 prescriptions (0.6%), but after its introduction, this number increased significantly to 21 out of 905 prescriptions (2.3%) (*p* = 0.0089)
Sonoda et al. [43]	An in-hospital PCS for hospitalized patients involved: (1) adding the label “renal” before the name of renally excreted drugs on the prescription; (2) adding the patient’s estimated kidney function level on the prescription; and (3) using a check sheet for the dosages based on kidney function for the target drugs. When a hospital prescription included the target drug, a check sheet based on the patient’s kidney function was automatically printed	The overall dosage error rate for the four target drugs was 26% before PCS implementation, which decreased significantly to 3% after PCS implementation (*p* < 0.001)

*CDSS* clinical decision support system, *DOAC* direct oral anticoagulant, *PCS* prescription checking system

Several studies have been conducted on pharmacists’ appropriate use of nephrotoxic drugs [45, 52–54]; in particular, there is a study on pharmacists using CDSSs to avoid administering nephrotoxic drugs to patients at risk of AKI. Dahmke et al. built a system to identify patients with decreased kidney function receiving triple whammy (TW) therapy, a combination of nonsteroidal anti-inflammatory drugs (NSAIDs), diuretics, angiotensin-converting enzyme inhibitors, or angiotensin receptor blockers and sent alerts to pharmacists [55]. In this study, 290 of the 21,332 hospitalized patients were prescribed TW, of whom 216 patients at risk of AKI were detected, and alerts were sent to pharmacists. Of these, pharmacists proposed an intervention to physicians in 94 cases and the proposal was accepted in 73 cases (77.7%). Thus, nephrotoxic drug-induced AKI may be prevented by pharmacists providing early intervention with the help of CDSSs for patients at risk for AKI, such as those receiving TW therapy.

## Effect of CDSSs on patient follow-up after administration of renally excreted or nephrotoxic drugs

This section discusses the utility of CDSSs for follow-up of patients administered renally excreted or nephrotoxic drugs. A worldwide meta-analysis of clinical epidemiological studies on AKI reported that the pooled incidence rates of AKI in hospitalized patients were 21.6% in adults and 23.2% overall, with a high incidence of 31.7% in intensive care units and 32.4% in patients with heart failure [13]. A retrospective cohort study at the University of Iowa Hospitals reported that 1527 of 11,311 hospitalized patients were exposed to high concentrations of nephrotoxic drugs for ≥ 1 day and subsequently developed AKI in 29% of cases; 22% of all hospitalized AKI events met the criteria for nephrotoxic AKI [11]. These reports indicate that the incidence of AKI during hospitalization and AKI due to nephrotoxic drugs is high and that it is important to closely monitor patients during the administration of renally excreted or nephrotoxic drugs. CDSSs can help detect the onset of AKI, slow its progression, and prevent the use of nephrotoxic drugs such as NSAIDs after AKI [56]. In the following section, this review describes the utility of CDSSs used by physicians for patients receiving renally excreted or nephrotoxic drugs (Table 3). McCoy et al. developed a system that sent interruption alerts to physicians when contraindicated or highly toxic medications needed to be avoided or adjusted when serum creatinine levels of hospitalized patients increased to 0.5 mg/dL or more within 48 h of prescribing at least one of 122 nephrotoxic or renally excreted drugs [34]. The rate of change or discontinuation per 100 events of medications included in the interruption alert within 24 h of an increase in creatinine levels improved from 35.2% before intervention to 52.6% after intervention (*p* < 0.001). Awdishu et al. created a system for outpatients and acutely ill adult patients with kidney impairment that monitored changes in kidney function in patients taking one of the targeted renally excreted or nephrotoxic drugs and recommended discontinuation or adjustment of drug dosage when necessary [25]. They then conducted a prospective cluster randomized controlled trial to compare physicians who received clinical decision support with those who performed the usual workflow. The rate of appropriate adjustment or drug discontinuation was significantly higher in the intervention group (10.9%) than in the control group (4%) (*p* < 0.0001). Pou et al. created a system to detect hospitalized patients who had an AKI event while taking nephrotoxic drugs and alert them to interruptions to determine whether the prescriber subsequently reduced the dose or discontinued the drug [37]. The proportion of patients whose nephrotoxic drugs were changed or discontinued increased to 78 out of 384 nephrotoxicity alerts (20%) in the control group and 154 out of 526 nephrotoxicity alerts (29%) in the intervention group (*p* < 0.01). Thus, CDSSs may be effective in allowing physicians to intervene in the changes in kidney function and AKI in patients administered renally excreted or nephrotoxic drugs.

**Table 3:** Summary of physicians’ approaches using CDSSs on patient follow-up after administration of renally excreted or nephrotoxic drugs

References	System features	Key results
McCoy et al. [34]	A system sent interruption alerts to physicians when contraindicated or highly toxic medications needed to be avoided or adjusted when serum creatinine levels of hospitalized patients increased to 0.5 mg/dL or more within 48 h of prescribing at least one of 122 nephrotoxic or renally excreted drugs	The rate of change or discontinuation per 100 events of medications included in the interruption alert within 24 h of an increase in creatinine levels improved from 35.2% before intervention to 52.6% after intervention (*p* < 0.001)
Awdishu et al. [25]	A system for outpatients and acutely ill adult patients with kidney impairment monitored changes in kidney function in patients taking one of the targeted renally excreted or nephrotoxic drugs and recommended discontinuation or adjustment of drug dosage when necessary	The rate of appropriate adjustment or drug discontinuation was significantly higher in the intervention group (10.9%) than in the control group (4%) (*p* < 0.0001)
Pou et al. [37]	A system detected hospitalized patients who had an AKI event while taking nephrotoxic drugs and alerted them to interruptions to determine whether the prescriber subsequently reduced the dose or discontinued the drug	The proportion of patients whose nephrotoxic drugs were changed or discontinued increased to 78 out of 384 nephrotoxicity alerts (20%) in the control group and 154 out of 526 nephrotoxicity alerts (29%) in the intervention group (*p* < 0.01)

*CDSS* clinical decision support system, *AKI* acute kidney injury

The usefulness of CDSSs for pharmacists in patients receiving renally excreted or nephrotoxic medications has also been studied [51, 57, 58]. McMullin et al. developed a system called DoseChecker at a large university hospital that targeted 35 renally excreted or nephrotoxic drugs and alerted pharmacists when dosage adjustments were necessary [58]. DoseChecker electronically screened 28,528 prescription orders and detected potential dosage issues in 2859 (10%). DoseChecker recommended a reduction in the daily dosage in 1992 (70%) prescription orders and an increase in the daily dosage in 867 (30%). Pharmacists contacted physicians about 1163 (41%) of the 2859 alerts, and physicians agreed to dosage adjustments in 868 (75%) cases. Schreier et al. developed a system that alerted pharmacists when it became necessary to reduce the dosage of these drugs due to changes in kidney function and have successfully used this system in clinical practice [51]. Thus, the use of the CDSS by pharmacists may improve the efficacy and safety of renally excreted or nephrotoxic drugs in patients receiving them.

## Conclusions

This review discussed the effectiveness, issues, and potential of CDSSs for physicians’ prescriptions and pharmacists’ prescription audits before hospitalized patients with kidney impairment are administered renally excreted drugs or nephrotoxic drugs, and the follow-up of patients receiving them. Although inappropriate prescriptions of renally excreted drugs due to alerts to prescribers were reduced, prescribers may have ignored interruption alerts. Therefore, the acceptance rate of alerts by prescribers can be improved by minimizing interruptions to the prescriber workflow, specifying only high-severity alerts, and automatically inputting the dosage, administration frequency, and administration duration according to kidney function when the prescriber selects a drug when entering a prescription. Prescription audits by pharmacists using electronic alerts from the CDSS and dosage confirmation sheets were effective in preventing overdosing of renally excreted drugs. In addition, pharmacist interventions for patients at risk of AKI using CDSS alerts may be useful in preventing a decrease in kidney function and the onset of AKI due to nephrotoxic drugs. Although the usefulness of CDSSs may be further improved in the future, further evaluation and improvement of CDSSs are required.

## Data Availability

No datasets were generated or analysed during the current study.

## Abbreviations

AKI: Acute kidney injury
BUN: Blood urea nitrogen
CKD: Chronic kidney disease
CDSSs: Clinical decision support systems
CI: Confidence interval
DOAC: Direct oral anticoagulant
eCCr: Estimated creatinine clearance
NSAIDs: Nonsteroidal anti-inflammatory drugs
PCS: Prescription checking system
ROC: Receiver operating characteristic
TW: Triple whammy
